# Migration Intent Among Early-Career Doctors in Pakistan: A Cross-Sectional Study of Influencing Factors at a Tertiary Care Hospital

**DOI:** 10.7759/cureus.110673

**Published:** 2026-06-11

**Authors:** Sadia Rehman, Shaikh Muhammad Owais Saeed, Muhammad Irfan Khattak, Hammad Ali, Gohar Awais, Sakina Shah Perver, Sania Nisar, Sana Younas

**Affiliations:** 1 Biochemistry, Bahria University Medical & Dental College, Karachi, PAK; 2 Medicine and Surgery, PNS Shifa Hospital, Karachi, PAK; 3 Nephrology, PNS Shifa Hospital, Karachi, PAK; 4 Research, The Aga Khan University, Karachi, PAK

**Keywords:** brain drain, emigration and immigration, interns and residents, pakistan, socioeconomic factors

## Abstract

Objective: The objective of this study is to assess the prevalence of migration intent among interns and residents at a tertiary care institution in Pakistan and to identify perception-based factors associated with this intent.

Methodology: A cross-sectional study was conducted at PNS Shifa Hospital using a structured, self-administered Google Form questionnaire (Google Inc., Mountain View, CA, USA). A total of 252 participants (house officers and postgraduate trainees) were recruited. Data were analyzed using IBM SPSS Statistics software, version 27 (IBM Corp., Armonk, NY, USA). Chi-square tests and binary logistic regression were applied to identify significant factors of migration intent.

Results: Out of 252 participants, 52% expressed a preference for postgraduate training abroad, with the United Kingdom (54.2%), the Middle East (16.8%), and the United States (15.3%) being the most preferred destinations. Key push factors included low salary (65.6%), poor working conditions (53.4%), and unemployment (46.6%). Logistic regression analysis demonstrated that perception of better quality of life (OR = 1.91, p = 0.015), improved training opportunities (OR = 1.77, p = 0.024), and a better working environment (OR = 1.95, p = 0.010) were significant predictors of migration intent.

Conclusion: This study provides institution-level evidence on migration intent among early-career doctors, highlighting the role of perception-driven factors in shaping career decisions. A substantial proportion of participants expressed an intention to pursue postgraduate training abroad, primarily due to perceived socioeconomic and training-related advantages abroad. These findings may inform institutional-level retention strategies and highlight areas for further multicenter and longitudinal research.

## Introduction

The term “brain drain" describes the migration of highly trained professionals to developed nations from their home country, frequently in search of a greater wage or standard of living [1]. Over the past 50 years, there has been a significant influx of healthcare workers to the United States of America (USA), the United Kingdom (UK), Canada, and Australia. Nine of the top 20 emigration-prone nations are found in Sub-Saharan Africa or the Caribbean; however, the leading sources of international medical graduates residing in these countries are from India, Pakistan, and the Philippines [2].

Despite facing a critical shortage of doctors and under-resourced healthcare facilities, Pakistan is the third-largest contributor of international medical graduates globally [3]. High levels of physician emigration have been reported in Pakistan; however, the intention to migrate among early-career doctors remains an important area of study. According to a survey conducted by the House of Commons in the year 2020, the National Health Service (NHS) staff had 13.8% of individuals who were not British in origin, and 29% had a foreign nationality. Regarding descent, 13.5% of them were of Asian descent, including 3,174 Pakistani doctors, and an even higher number (5,399 of the total 19,000) had their primary medical qualification from Pakistan.

Similarly, in the USA, out of the 24.7% of international medical graduates registered with the American Medical Association Physician Masterfile, 29.7% were Pakistani [4]. Migration intent is a significant concern in Pakistan, as it may reflect a potential future loss of human capital if translated into actual migration [5]. Pakistan faces concerns regarding the potential loss of healthcare professionals, making it important to understand the factors influencing migration intentions. Various studies have identified a number of factors that are responsible for doctors' preference for postgraduate training abroad. For instance, a study conducted in two medical colleges in Pakistan found that the majority of students believe inadequate salaries and long working hours lead to poor patient management and insufficient training, making them key push factors for migration [6]. Other traditional factors driving doctors out of the country include inflation, rising capital share in gross domestic product, and unemployment [7]. Finding training positions is becoming increasingly difficult for recent graduates, and at least twenty individuals are currently waiting for one training spot. This has made young doctors quite resentful, and as a result of this, they want to pursue postgraduate training abroad [8]. Insecurity, economic problems, lack of social support, family ties, peer pressure, favoritism, terrorism, harassment, and religious and political views of doctors are some of the other factors contributing to migration intent in Pakistan [9].

The incentives attracting doctors to leave Pakistan include higher salaries, superior training quality, job satisfaction, improved quality of life, more career opportunities, a better working environment, the aspiration to settle overseas, and more effective healthcare management [10]. Existing literature suggests that physician emigration may have implications for health system performance; however, our study does not directly evaluate these institutional effects, compounding the effects of existing challenges. In addition to the threat posed by population growth, acute poverty, and a high burden of infectious and non-infectious diseases, Pakistan currently has one of the worst rates of maternal and perinatal deaths in the world [6]. To regulate the movement of healthcare professionals across nations, the government and other organizations must adopt a strategic approach.

This study explores migration intent among early-career doctors, focusing on their preference for postgraduate training abroad. It aims to estimate the prevalence of migration intent and identify perception-based factors associated with this intent among early-career doctors. The findings offer valuable insights for global collaboration on addressing medical workforce migration.

## Materials and methods

This study employed a quantitative cross-sectional design and was conducted over the period of nine months at PNS Shifa Hospital, a tertiary care teaching hospital in Karachi, Pakistan. Prior to commencement, ethical approval was obtained from the Institutional Ethics Committee of the hospital on May 13, 2024 (approval Number: ERC/2024/MED/93). All procedures followed ethical research standards, which included obtaining informed consent from participants and ensuring strict confidentiality and anonymity of data.

The sample size was calculated using the OpenEpi Sample Size Calculator (version 3; Dean, A. G., Sullivan, K. M., & Soe, M. M. (2013)). OpenEpi: Open Source Epidemiologic Statistics for Public Health (Version 3.01). www.OpenEpi.com), based on a population of 394 individuals representing the eligible population within the study institution. A hypothesized frequency of 50% for the primary outcome was assumed to maximize variability and ensure conservative estimation. Using a ±5% margin of error and a 95% confidence level, the minimum required sample size was determined to be 252 participants, deemed sufficient to provide statistically reliable estimates within the study population.

A self-administered, structured questionnaire was developed using Google Forms (Google Inc., Mountain View, CA, USA) to ensure clarity, relevance, and ease of completion. It was designed after a thorough literature review and team discussions to enhance content relevance and face validity. The questionnaire assessed demographic characteristics, career aspirations, postgraduate training preferences, and factors influencing migration decisions. It consisted mainly of closed-ended questions, supplemented by Likert-scale and multiple-choice items, allowing for standardized responses and nuanced insights. The survey was pilot tested before full deployment to assess clarity and comprehensibility.

Participants were systematically selected through consecutive sampling; all individuals meeting the predefined inclusion criteria were screened and enrolled sequentially until the target sample size was reached. Inclusion criteria specified house officers and postgraduate trainees employed at PNS Shifa Hospital during the time of the study who gave consent. MBBS students, consultants, and those declining consent were excluded. All eligible participants received detailed information about the study’s purpose, procedures, and their rights. Written informed consent was obtained, emphasizing confidentiality, voluntary participation, and the right to withdraw at any time without repercussions.

Data were entered and analyzed using IBM SPSS Statistics software, version 27 (IBM Corp., Armonk, NY, USA). Descriptive statistics, including frequencies and percentages, summarized demographic and survey data. To explore associations between categorical variables and identify factors of migration intent, chi-square tests were used. Perception-based variables (e.g., quality of life, training, and working environment abroad) were conceptualized as cognitive evaluations influencing migration intent rather than as equivalent constructs. Odds were calculated by univariate binary logistic regression. Multivariate binary logistic regression was applied for variables significant on univariate analysis to get adjusted odds ratios.

## Results

The study included 252 participants, with 63.5% females and a mean age of 26.47 ± 3.13 years. The sample comprised an equal number of house officers and residents (50% each). Notably, 52% (131 participants) expressed a preference to migrate abroad for postgraduate training (Table 1).

**Table 1:** Demographic characteristics of the participants

Variable	Category	N (percentage)
Gender	Male	92 (36.5%)
	Female	160 (63.5%)
Age group	≤ 28 years	201 (79.8%)
	> 28 years	51 (20.2%)
Current occupation	House Officer	126 (50%)
	Resident	126 (50%)
Postgraduate training pathways	Pakistan	121 (48%)
	Abroad	131 (52%)

**Figure 1:**
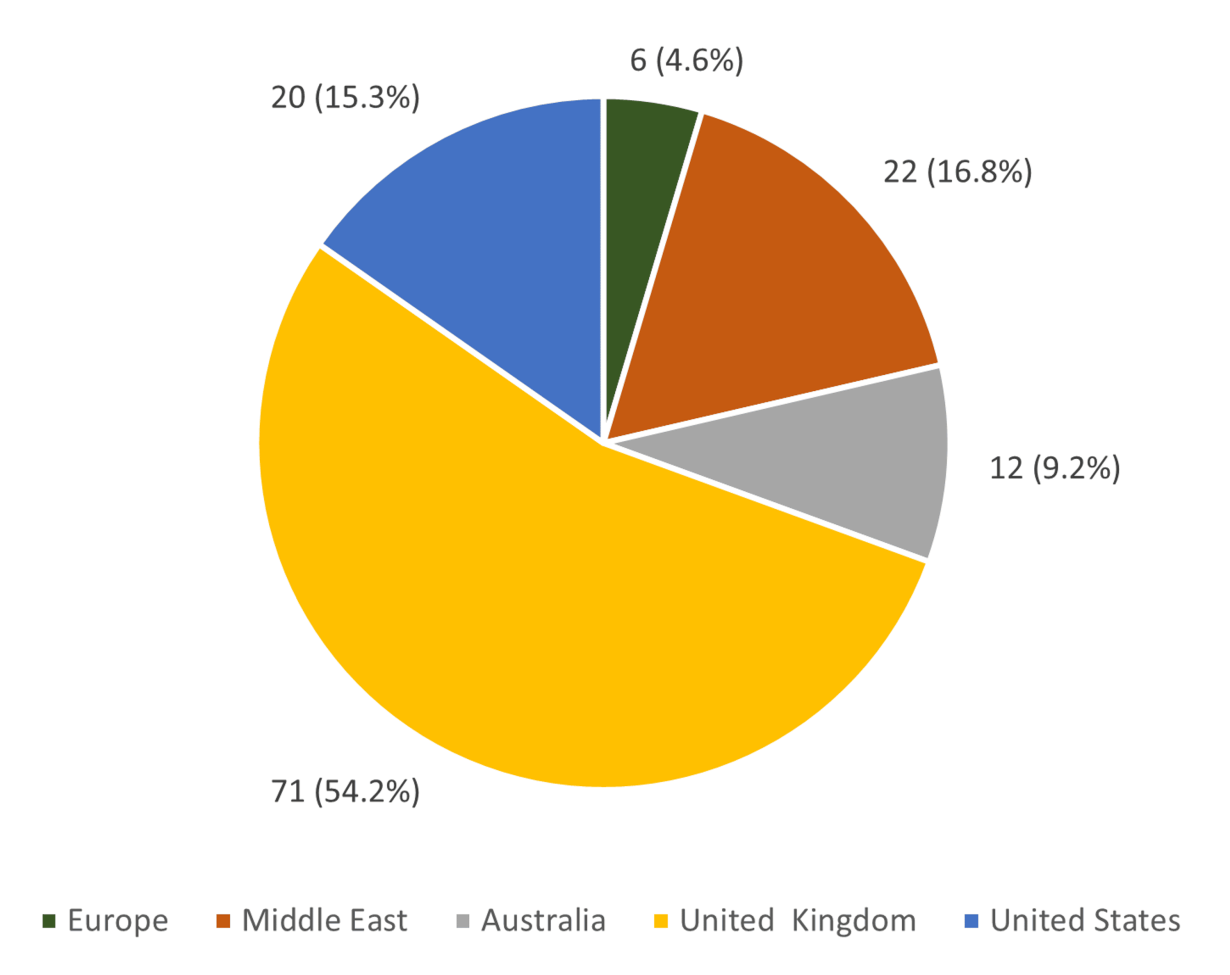
Country of choice for migration according to the participants

Among those preferring postgraduate training abroad, the UK was the top choice, selected by over half of the respondents. The Middle East and the USA followed, with fewer choosing Australia and Europe (Figure 1).

**Figure 2:**
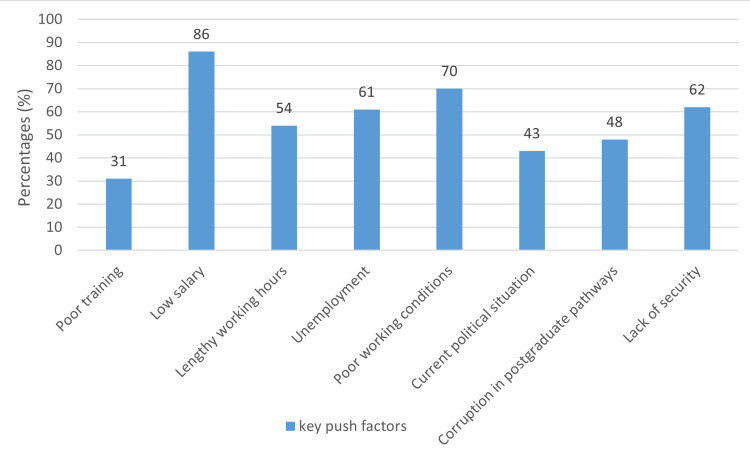
Key push factors for migration (n = 131)

A majority of the participants cited low salary and poor working conditions as primary reasons for going abroad, with more than half also expressing concern about personal safety. Other contributing factors included unemployment, long working hours, and corruption in postgraduate pathways. The political climate and dissatisfaction with local training were also noted by several respondents (Figure 2).

We found a significant association between migration preferences with agreement that doctors who live abroad have a better quality of life (p=0.015/OR=1.915), agreement that doctors have better training abroad (p=0.024/OR=1.77), and agreement that doctors have a better working environment abroad (p=0.010/OR=1.951) (Table 2).

**Table 2:** Perceptions of doctors who intend to migrate abroad (n=131) OR: odds ratio; CI: confidence interval

Perceptions	Percentage agreed (%)	p-value	OR (95% CI)
Doctors abroad have a better quality of life	71.8	0.015*	1.915 (1.134-3.232)
Doctors earn more abroad	60.3	0.692	1.107 (0.670-1.830)
Doctors are more secure abroad	51.9	0.092	1.533 (0.931-2.523)
Doctors receive better training abroad	57.3	0.024*	1.777 (1.079-2.928)
Doctors learn from modern technologies abroad	57.3	0.819	0.943 (0.572-1.556)
Doctors have a better working environment abroad	67.9	0.010*	1.951 (1.170-3.254)

## Discussion

This study explores the perceptions of early-career doctors in Pakistan regarding postgraduate training and migration abroad, while focusing on perception structures rather than objective migration determinants. The findings suggest that migration intentions are shaped not only by aspirations for career advancement but also by perceptions surrounding training quality, professional development opportunities, working conditions, and long-term career stability. These patterns are consistent with existing literature describing physician migration as a multifactorial phenomenon influenced by both systemic push factors and perceived opportunities in higher-income healthcare settings.

Around 32,879 physicians graduate each year in Pakistan, 40% of whom leave for better opportunities abroad [11]. Previous studies have highlighted the migration of international medical graduates from low- and middle-income countries to high-income nations such as the USA, the UK, Canada, and Australia primarily for better living standards, advanced training, and financial stability [12]. Our findings reflect a similar trend, with a clear preference for Western, English-speaking countries, particularly the UK and the US. However, a notable divergence is the rising interest in Middle Eastern countries as postgraduate training destinations. This shift suggests that younger doctors are now considering regional alternatives offering competitive opportunities, easier relocation, and cultural or geographical proximity. Moreover, growing saturation and competition in traditional Western destinations may be prompting a reconsideration of preferences. To enhance analytical clarity, the factors influencing migration intent identified in this study can be interpreted using a push-pull framework. Push factors represent conditions within the home country that encourage doctors to consider leaving, while pull factors represent perceived advantages of training and working abroad. These factors can further be categorized into macro-level (structural), meso-level (institutional), and micro-level (individual) influences. In addition to the push-pull framework, these findings are interpreted in light of human capital theory and expectation-reality perspectives to provide a more comprehensive understanding of migration intent.

In 2023, Meo Ayoub S. et al. reported that around six million highly qualified and professional individuals have left the country over the last five decades [13]. Using a push-pull framework, the factors influencing migration intent can be categorized into macro-level (socioeconomic and political conditions), meso-level (institutional and workplace factors such as salary and training environment), and micro-level (individual perceptions of quality of life and opportunities abroad). Together, these findings indicate that migration intent is shaped by the interaction of structural, institutional, and individual-level influences [14]. Our findings also highlight several pressing local challenges influencing doctors’ intention to migrate. A significant proportion of respondents identified low salary (86%) and poor working conditions (70%) as primary motivations. Concerns regarding personal safety were raised by a substantial portion of the respondents (62%), indicating that this has become a significant factor influencing their intent to migrate. Other notable factors included unemployment (61%), excessive working hours (54%), corruption in postgraduate pathways (48%), a volatile political environment (43%), and growing discontent with local training standards (31%), all of which indicate commonly reported concerns among early-career doctors in our sample. This contrasts with a 2024 study conducted by Meo Ayoub S. et al., which identified instability in the sociopolitical environment in Pakistan as the leading cause of the migration intent [15].

Within the push-pull framework, micro-level perception factors were found to be significant in our study. Respondents who believed that doctors overseas enjoy a better quality of life (OR = 1.91, p = 0.015), receive higher-quality training (OR = 1.77, p = 0.024), and work in a more favorable environment (OR = 1.95, p = 0.010) were more likely to express an intention to pursue postgraduate training abroad. These perceptions were significantly associated with migration intentions among early-career doctors. This reflects the expectation-reality discrepancy framework, where anticipated benefits of migration may not always align with actual experiences. A nationally representative study conducted in the United States among 1,890 primary care physicians found that international medical graduates were significantly less likely to report career satisfaction compared to their US-trained counterparts (75.7% vs. 82.3%; p = 0.005) [16]. This difference persisted after adjusting for relevant variables (adjusted OR = 0.62; 95% CI: 0.43-0.90). The study further identified factors such as income level, specialty, and practice setting as contributors to overall satisfaction. These findings underscore the possibility that, despite optimistic perceptions, the realities of working abroad may not always fulfil professional expectations, especially for international medical graduates navigating unfamiliar healthcare systems. These findings can also be interpreted through a human capital perspective, where migration decisions are viewed as investments aimed at maximizing future professional and economic returns. Perceptions of better training opportunities, improved working environments, and higher quality of life abroad may therefore reflect anticipated gains in skills, career advancement, and long-term benefits.

While our study offers valuable insights into the motivations behind medical migration, there is a pressing need to further explore the real-world experiences of doctors who have already relocated. Future research should further explore whether migration intentions translate into actual migration behavior. This structured framework highlights that migration intent is not driven by a single factor but by the interaction of macro-level conditions, meso-level institutional environments, and micro-level individual perceptions. Such research may provide further insight into the relationship between expectations and experiences of doctors working abroad. Although physician availability is an important component of healthcare delivery, this study does not directly examine its relationship with maternal or perinatal outcomes.

These findings suggest potential areas for institutional consideration, including identifying strengths and opportunities within the healthcare system. These findings may help inform discussions on workforce retention and postgraduate training environments and create an environment where doctors feel motivated to stay and serve their communities. Strengthening support for healthcare professionals may contribute to improved workforce retention and system stability. This study measures migration intention, not actual migration behavior, and therefore does not directly quantify physician outflow. Additionally, given that both the predictors and outcome were perception-based, there is potential for conceptual overlap, which should be considered when interpreting these associations. Additionally, as a cross-sectional, single-center study based on self-reported data, causal relationships cannot be established, and generalizability may be limited. The sample is representative of early-career doctors within the institution but may not reflect national trends. Formal psychometric validation of the questionnaire was not performed, which is a limitation of the study.

## Conclusions

A considerable proportion of early-career doctors in this institution reported an intention to pursue postgraduate training abroad. This intention was significantly associated with perceptions of better quality of life, training opportunities, and working environments abroad. As the study measures intention rather than actual migration behavior, further longitudinal and multicenter research is needed to examine whether these intentions translate into emigration. Data from such studies may provide further insight into factors related to workforce retention and healthcare system dynamics.
